# Nonfactor Therapies in Hemophilia A: An Essential Drug Entity

**DOI:** 10.7759/cureus.70763

**Published:** 2024-10-03

**Authors:** Rahul U Ramachandran, Vishnu Sharma, Shailendra P Verma

**Affiliations:** 1 Pediatrics, National Health Mission (NHM) Kerala, Trivandrum, IND; 2 Clinical Hematology, Sawai Man Singh (SMS) Medical College, Jaipur, IND; 3 Hematology and Oncology/ Clinical Hematology, King George's Medical University, Lucknow, IND

**Keywords:** cost-effective, emicizumab, hemophilia a, rare disease, therapy

## Abstract

Inequity in the access to effective therapies for rare diseases, such as hemophilia A, is a significant concern in resource-limited settings. Including necessary medications in the essential drug list can ensure that populations have access to safe and affordable treatments. Emicizumab, a nonfactor therapy, addresses many of the challenges associated with traditional factor replacement for hemophilia A, including frequent administration and the risk of inhibitor development. Studies in India show that emicizumab is cost-effective and clinically beneficial. The inclusion of emicizumab in the National Health Mission’s (NHM) Essential Drug List (India) would improve treatment access and adherence, reduce healthcare burden, and improve outcomes for patients, particularly those with high bleeding rates and poor accessibility to care. Moreover, it will ensure the continuity of therapy for all existing patients who have been on emicizumab supported by the NHM in the last four years. This review aims to consolidate evidence regarding the current status of care and the benefits of emicizumab in India to highlight the importance of the addition of emicizumab to NHM’s Essential Drug List.

## Introduction and background

Hemophilia is a rare genetic disorder classified mainly into two types, hemophilia A and hemophilia B, and results from a deficiency or complete absence of clotting factors. Spontaneous bleeding in joints and muscles can occur in severe patients with hemophilia (PwH). Repeated bleeding episodes can result in irreversible joint damage, potentially leading to disability [1,2]. Hemophilia A primarily impacts individuals by causing mobility challenges, unexpected bleeding episodes, pain, and uncertainty in their daily activities [3]. Patients with hemophilia (PwHA) tend to have a poor quality of life, affecting physical, social, psychological, and economic well-being [4]. The associated burden of hemophilia A can be more severe than that of hemophilia B [5].

The conventional management of hemophilia A includes episodic (on demand) or prophylactic factor replacement. Such treatment is usually started at an early age and requires multiple intravenous access weekly. On-demand factor administration for acute bleeding is also needed [6]. Prophylaxis facilitates sustained hemostatic control for preventing spontaneous and unpredictable bleeding among PwHA. Clotting factor concentrates (CFCs) derived from human plasma or produced using recombinant technology from cell cultures are typically used for prophylaxis. However, the short half-life, need for intravenous administration, and potential for inhibitor formation pose considerable limitations to their use [7]. In addition, the lack of access to treatment remains a major factor in India that compromises the quality of life of patients [8]. One of the key strategies for improving the quality and equity of healthcare access is ensuring that a select group of essential drugs is available within functioning health systems, e.g., the National Health Mission (NHM) (India )[9].

In 2015, bleeding disorders received attention under the NHM, reflecting an increased awareness and commitment to addressing these serious conditions. In 2024, the NHM helped to improve care by including hemophilia treatments in the Essential Drug List (D. O. No. NHSRC/13-14/Q1/01/DPSEPF-2) [10]. This inclusion marked a pivotal step in increasing access to vital treatments for PwHA, ensuring that essential factor replacement therapies were more readily available in public healthcare facilities.

Now, newer agents are making treatment easier and helping to reach patients in remote regions. The nonfactor replacement therapies are being adopted worldwide and even in many states in India. Nonfactor hemophilia therapies, such as emicizumab, were not included in the Essential Drug List. Emicizumab, a nonfactor therapy for hemophilia A, has been recognized for its significant benefits over traditional factor replacement therapies. It is included in the Central Government Health Scheme (CGHS) life-saving drug list, acknowledging its critical role in managing hemophilia A [11]. Emicizumab offers several advantages, including subcutaneous administration, less frequent dosing, and a reduced risk of developing inhibitors [6,12]. This review assesses current issues and evidence outlining the advantages to outline the value of the inclusion of emicizumab in the NHM’s Essential Drug List.

## Review

Hemophilia A and its impact

Hemophilia A is the most common X-linked hereditary disorder of hemostasis occurring due to a mutation in the F8 gene, which encodes coagulation factor (F) VIII, essential for normal blood clotting. It manifests as spontaneous or trauma-induced hemorrhagic episodes in patients, progressing to chronic disability and premature mortality in sub-optimally treated or untreated patients [8]. Bleeding secondary to minor trauma is seen in mild (factor level >5% and <40% of normal; FVIII 0.05-0.4 IU/mL) and moderate (factor level 1%-5% of normal; FVIII 0.01-0.05 IU/mL) types, but spontaneous bleeding occurs in severe (factor level <1% of normal; FVIII <0.01 IU/mL) cases [7]. PwHA constitutes nearly 70% of patients with bleeding disorders and 80% of all hemophilia cases [13,14]. In 2011, the reported number of PwHA was 11,586, with an estimated prevalence of around 50,000 [8]. However, according to a report by the Indian Council of Medical Research in 2019, there were an estimated 80,000-100,000 severe PwHA in India, with only 19,000 registered with the Hemophilia Federation of India [15]. The lack of awareness and diagnostic facilities and the high cost of tests contribute to the underreporting of bleeding disorders in India.

**Figure 1:**
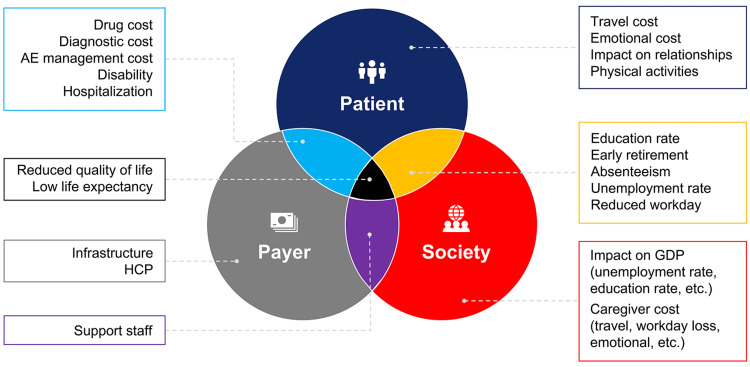
Socioeconomic cost of hemophilia A AE: adverse event; HCP: healthcare provider; GDP: gross domestic product

Hemophilia A is linked to a considerable burden on individuals, healthcare systems, and society. The development of neutralizing antibodies, known as inhibitors, further increases the socioeconomic burden [16]. Hemophilia A has been reported to be associated with negative impacts on employment and disability rates, absenteeism from work or school, perceived impact on education or career, and social functioning [1]. Frequent bleeds and pain can result in increased activity impairment and work productivity loss. However, such issues can be prevented by lifelong prophylaxis and high therapy adherence [17]. It has been reported that the out-of-pocket (OOP) expenditure of PwHA ranged from 1.5% to 12% of monthly family income. However, that estimate was based on the report that only one in four bleeding episodes was treated with factor concentrate [18]. Even with government programs to reduce OOP expenditure, the major barrier to care has been reported to be the difficulty in commuting during active bleeding episodes [19]. The socioeconomic implications of hemophilia A on patients, payers, and society are outlined in Figure 1** **[1,16-19].

Hemophilia A: treatment and challenges

**Figure 2:**
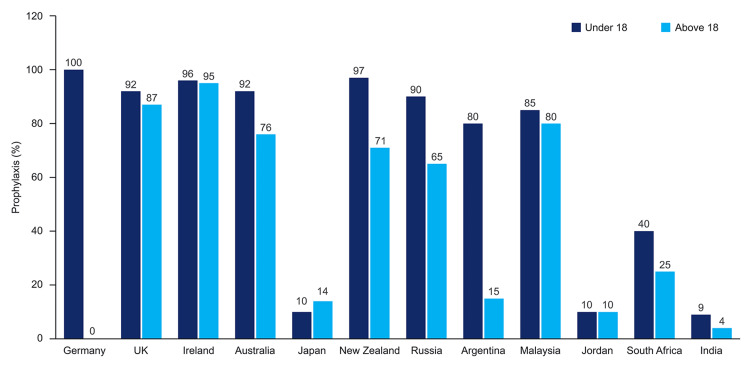
Percentage of patients with hemophilia receiving prophylaxis across countries Source: Oldenburg J [21]

The primary goal for the treatment of hemophilia A is to prevent and treat bleeding by replacing the deficient clotting factor, preferably in a comprehensive care setting. Infusion of FVIII concentrates is carried out episodically or prophylactically to prevent bleeding and its deleterious effects on joints and muscles [20]. Achieving successful long-term outcomes in PwHA lies in implementing effective prophylaxis to prevent joint bleeding. Effective prophylaxis must consider available resources, bleeding triggers, and the number of acceptable bleeds. Primary prophylaxis aims to prevent joint damage as once joint damage occurs, it progresses over the lifetime, even when no further bleeds occur in affected joints [21]. However, the adoption of prophylactic therapy is currently about 4% in India, compared to 20% in some other developing countries and 80%-90% in developed countries (Figure 2) [7,22].

A study from Kerala reported a death rate of 32.9 per 1,000 among PwHA, with none of them on prophylaxis or home therapy [23]. Such statistics substantiate the need for improved access to prophylaxis, also called regular replacement, to avoid preventable deaths.

The lack of access to treatment contributes to disability and severely compromises the quality of life of patients [7,8]. In addition, without access to treatment, PwHA experience frequent absenteeism from school and work, as physical activity is limited due to the extreme pain and discomfort associated with the hemorrhagic episode. The management of hemophilia A has primarily included prophylactic factor replacement with additional on-demand factor administration for acute bleeding episodes. This requires intravenous injections three times a week, with additional intravenous injections following a bleed. This is because factor replacement prophylaxis leads to fluctuations in plasma concentration of the clotting factors in the form of peak activity followed by low trough levels, which are the danger period for breakthrough bleeds [24]. A major complication with replacement factor therapy is the development of neutralizing antibodies, known as inhibitors. Such inhibitors develop in about 30% of severe PwHA (in addition to 5% of patients with mild and moderate hemophilia A) and can complicate disease management, impacting quality of life and prognosis [25]. Testing for inhibitors requires expert laboratory personnel and is time-consuming. These tests are not widely accessible. Once PwHA develop inhibitors, their access to hemophilia care is further significantly hindered by a necessary change in treatment. PwHA with inhibitors require management with more complex regimens with bypassing agents, such as recombinant factor VIIa (rFVIIa) and activated prothrombin complex concentrates (aPCCs). In addition, the experience of hematologists in India with immune tolerance induction (ITI) to eradicate inhibitors is limited [7,26]. Frequent intravenous injections cause pain and poor venous access, with multiple injections contributing toward complications, such as vein damage, scarring, and compartment syndrome, leading to poor compliance [6,27]. Patients often have to travel long distances to avail of treatments, such as intravenous factors or bypassing agents [23]. Another limitation is the short half-life of these factors or bypassing agents, necessitating frequent administration, usually one to three times a week, even with the extended half-life factor [24]. Gene therapy for hemophilia A has shown promising potential [28]. Recently, India has conducted the first human clinical trial of gene therapy for hemophilia A at the Christian Medical College, Vellore [29]. However, much effort is needed to make such an approach feasible and cost-effective for broader use in India.

Nonfactor therapies

The introduction of nonfactor drug products has shown significant potential in improving the management of hemophilia A. Emicizumab is a nonfactor therapy that acts as a bridge between FIXa and FX, enhancing coagulation [30]. The efficacy and safety of emicizumab have been demonstrated in HAVEN clinical trials involving both children and adults with hemophilia A, both with and without inhibitors, using weekly, biweekly, or monthly subcutaneous dosing [31]. The studies report hemostatic efficacy and safety with low rates of breakthrough bleeding episodes. The World Federation of Hemophilia (WFH) advises that PwHA begin home therapy with emicizumab following proper training in subcutaneous injection techniques. Emicizumab offers several advantages, including an easier route of administration, eliminating the need for central venous access devices (which is particularly beneficial for infants), infrequent dosing intervals of once or twice a month, and without the peaks and troughs as associated with CFCs, resulting in zero bleeds and even resolution of target joints, which are frequent in patients [7]. In addition, routine monitoring is not required with emicizumab in the clinical setting, thereby reducing the burden on healthcare resources and making it easier for patients from remote locations [32].

Cost-effectiveness of emicizumab

India's healthcare system is a mix of both public and private sectors. The public healthcare system is largely governed by the Ministry of Health and Family Welfare and aims to provide Universal Health Coverage (UHC) through various government initiatives. However, despite these efforts, access to quality healthcare remains uneven, particularly in rural and underserved regions. Other challenges include limited health insurance coverage, insufficient public healthcare funding, and a fragmented healthcare system. The high OOP expenses for healthcare services can be a major burden for many Indians [33]. Emicizumab has been evaluated for its cost-effectiveness in treating hemophilia A in India through several studies, highlighting its economic advantages compared to traditional therapies. In the study by Krishnamoorthy et al., a cost-utility analysis using a Markov model showed that prophylactic emicizumab therapy is a cost-saving intervention for severe PwHA with inhibitors. The analysis revealed negative incremental cost-utility ratios (ICURs) when compared to rFVIIa and aPCCs, indicating that emicizumab is both less costly and more effective in India [34]. Another study by Kar et al. employed an adaptive health technology assessment (aHTA) methodology to assess emicizumab prophylaxis. The findings demonstrated potential cost savings from the health system perspective using various adjustment methods. The study concluded that emicizumab prophylaxis could lead to significant cost savings and is feasible for implementation in India [35]. Seth et al. conducted a cost-effectiveness analysis comparing emicizumab prophylaxis to on-demand therapy and various doses of FVIII prophylaxis. The study found that emicizumab was cost-effective, especially when compared to high-dose prophylaxis, with favorable incremental cost-effectiveness ratios (ICERs) per quality-adjusted life-year (QALY). Probabilistic sensitivity analysis indicated a high probability of emicizumab being cost-effective at willingness-to-pay (WTP) thresholds of two to three times per capita gross domestic product (GDP) [36]. As per the WHO, the WTP threshold for health interventions typically ranges from one to three times the per capita gross domestic product (GDP) [37]. For the year 2023, given that India's per capita GDP was approximately USD 2,400, the WTP threshold would be between USD 2,400 and USD 7,200 [38]. These studies collectively highlight that emicizumab prophylaxis is a cost-effective and sustainable option for managing hemophilia A in India, offering substantial economic and clinical benefits over traditional bypassing agents and FVIII prophylaxis.

The role of the Essential Drug List

Inequity in access to effective therapies remains an issue of concern for individuals living with rare diseases [39]. The concept of essential medications plays a crucial role in healthcare systems worldwide, ensuring that populations have access to safe, effective, and affordable medications for priority health needs. The WHO has been a key advocate for essential medications, issuing its first Model List of Essential Medications (EML) in 1977 and regularly updating it to reflect evolving healthcare needs (currently the 23rd list; 2023) [40]. Essential medications are identified based on their relevance to public health and evidence of efficacy, safety, and cost-effectiveness. By applying these criteria, the WHO aims to create a comprehensive and evidence-based model list of essential medications that can guide countries in prioritizing the procurement, distribution, and use of medications that are essential for promoting public health and improving healthcare outcomes globally [41].

In India, the NHM serves as a cornerstone for various healthcare initiatives launched by the government [42]. The overarching aim of the NHM is to ensure universal access to equitable, affordable, and quality healthcare services [42]. In addition to other activities aiming to promote well-being, it plays a crucial role in ensuring the availability of essential drugs in public healthcare facilities. The NHM provides financial support to states and union territories to supply essential medications in public health facilities. This initiative aims to make healthcare more accessible, affordable, and of high quality for all individuals utilizing public health services. To streamline the procurement, distribution, and management of essential drugs, NHM has recommended the creation of an Essential Drug List for public healthcare facilities. The NHM’s Essential Drug List specifies the essential drugs that should be available at various levels of healthcare facilities, including sub-health center-health and wellness centers (SHC-HWCs), primary health center-health and wellness centers (PHC-HWCs), community health centers (CHCs), and district hospitals (DHs). In addition to ensuring the availability of essential drugs, the NHM emphasizes the importance of rational drug use and prevention of wastage. Measures such as prescription audits, dissemination of standard treatment guidelines (STGs), and the establishment of the drugs and vaccine distribution management system (DVDMS) are implemented to monitor and regulate the distribution and utilization of drugs in public healthcare facilities. By promoting the use of essential drugs and using effective strategies to monitor drug distribution, the goal of enhanced quality healthcare services and a marked reduction in OOP expenses for patients can be achieved.

The inclusion of emicizumab in the NHM’s Essential Drug List will ensure broader access to many more PwHA, particularly those in remote or difficult-to-access regions, after serious bleeds, high bleeders and those with inhibitors. This effective nonfactor hemophilia therapy will contribute to better joint outcomes with less frequent injections and improved quality of life, all of which are unmet needs of PwHA in India. By reducing the frequency of hospital visits, the need for complex medical interventions, and the need for screening for inhibitors, emicizumab can help improve outcomes of hemophilia A management and enhance the overall efficiency of healthcare delivery.

Case study: the Ashadhara Project (Kerala)

The guidelines for the management of hemophilia published by the WFH serve as a reference for defining care protocol for PwH. It offers a means to bridge the gap between treatment practices and care around the world. In addition, they provide a set of practical recommendations to improve the quality of care in resource-limited settings, aiming at effective clinical interventions and decreasing variation in clinical practice. To provide access to treatment at par with global standards in hemophilia, the state of Kerala has adapted the WFH model for care delivery.

There are 2,094 registered PwH in the state with 1,658 PwHA and 436 PwHB. Hemophilia has been addressed on a case-based approach through tertiary care centers in the state for a long time. Since 2012, treatment with factors has been made available free of cost under Kerala Community Pharmacies of Kerala Medical Supplies Corporation Limited. Since 2014, an effort has been initiated through DH, Aluva to set up a comprehensive Hemophilia Treatment Center that provides a range of services, including factor level assessment, inhibitor status, management (including complications), rehabilitation, counseling, and capacity building for support. In 2020, the Government of Kerala launched the Ashadhara project to improve the quality of life and drive better patient outcomes. This project aims to improve access to care through a comprehensive decentralized care delivery strategy. The objective of the Aashadhara project is to facilitate prompt on-demand care and prophylactic care for children under the age of 18 for hemophilia and establish a patient-centric care delivery model to enhance the quality of life for PwH. The challenges faced by PwH are outlined in Table 1.

**Table 1:** Challenges faced by patients with hemophilia OOP: out of pocket, PwH: patients with hemophilia Sources: [1,16–19]

Challenge	Description
Lack of awareness	Lack of awareness and misconceptions among patients about the disease, treatment options, and services is a major challenge, leading to an overburdened health system due to patient aggregation at a few centers, resulting in delayed care delivery.
Orthopedic complications and disability	The long-term consequences of repeated joint bleeds with sub-optimal treatment are the development of chronic and progressive joint damage and disability.
Prevalence of inhibitors	One of the complications of replacement therapy is the development of alloantibodies called inhibitors. The reported prevalence of patients with inhibitors is between 5% and 7%, with the risk of inhibitor development being higher in patients with severe rather than mild or moderate hemophilia.
Health system impact	Disease-specific needs and the life-long nature of the disease lead to high health system consumption. This, coupled with minimal capacity and capabilities for diagnosis, monitoring, and treatment of hemophilia, leads to poor patient experience and quality of care.
Access to treatment	Management of hemophilia requires repeated hospital admissions. Lack of access to district-level treatment facilities involves extensive travel for the patient and results in further aggravation of the patient's condition. Such issues, along with extensive OOP expenditure, significantly affect the quality of life and well-being of patients and family members.
Impact on schooling and employment	Without access to treatment, PwH experience frequent absenteeism from school and work, as physical activity is limited due to the extreme pain and discomfort associated with the hemorrhagic episode.

Given the challenges across the care continuum, from awareness to rehabilitation, and associated social costs in terms of morbidity and mortality, schooling, and employment, the Ashadhara project builds upon a state-wide strategy with components that are explained in Table 2.

**Table 2:** Components of the state-wide strategy used by the Ashadhara project to address challenges across the care continuum

Component	Details
Strengthening health system	Upgrading infrastructure of hemophilia treatment centers and District Day Care Centers (DDCCs) to Centers of Excellence for diagnosis, treatment, and patient support services. Capacity building of healthcare workers to provide comprehensive care, including health literacy, patient management, and patient/caregiver counseling.
Prompt on-demand treatment	Decentralizing care delivery to district and sub-divisional hospitals, with multidisciplinary support provided by tertiary care facilities for complex cases. This reduces travel time and long-term impacts, such as structural deformities and disabilities.
Risk-stratified prophylactic care	Establishing prophylactic care for patients under 18 years of age and facilitating access to innovative therapies, such as emicizumab, through a subject matter expert committee evaluation. This approach sets a precedent for outcome-based management, resulting in cost-effective and substantial budgetary savings.

Among the 2,094 registered PwH, 1064 are categorized as severe. The risk-stratified prophylactic care model has categorized these patients based on their needs, with 316 patients on prophylaxis. Of these, 210 receive factor prophylaxis, and 106 receive emicizumab prophylaxis. The patient archetypes receiving emicizumab prophylaxis include inhibitor patients under 18 years old (43.4%), non-inhibitor patients (36.8%), patients under three years of age with venous access issues (26.4%), patients aged three-18 years with sub-optimal response to biweekly factor prophylaxis: 36.8%); and inhibitor patients over 18 seeking episodic treatment (19.8%).

The diversity of patient archetypes receiving emicizumab showcases the ability of the Ashadhara project to identify the right patients for innovative therapies, demonstrating a value-based care model. Kerala's health system has enabled equitable access to innovative therapies through protocol-driven management and health system strengthening, resulting in improved patient outcomes and quality of life for PwH. The success of this project highlights the importance of making emicizumab available. Such inclusion would ensure broader access to this life-saving therapy, further enhancing the quality of care and outcomes for PwH across India.

Recommendations for policy and practice

The recent inclusion of hemophilia drugs in the NHM’s Essential Drug List is a positive effort to ensure availability; however, the inclusion of emicizumab has been overlooked (D. O. No. NHSRC/13-14/Q1/01/DPSEPF-2) [10]. Emicizumab addresses the current challenges presented by conventional clotting factor replacement with the potential to improve treatment adherence, making it a suitable candidate for inclusion in the NHM’s Essential Drug List. Such a step will help address the existing unmet needs in hemophilia A care in India. Patients who will benefit the most from the inclusion of emicizumab in the NHM’s Essential Drug List include both patients with and without inhibitors. This includes non-inhibitor patients with high annual bleeding rates, those at risk of rebleeding after severe bleeds such as intracranial or psoas bleeds, individuals living far from health centers, those with poor venous access, and those lacking adequate monitoring or healthcare resources. In addition, PwHA with inhibitors will greatly benefit from this effective and manageable treatment option, which can significantly improve their quality of life and reduce the burden on healthcare systems. The Ashadhara project, launched by the Government of Kerala, aims to improve access to comprehensive hemophilia care through a decentralized and patient-centric approach [43]. By leveraging a risk-stratified prophylactic care model and introducing innovative therapies, such as emicizumab, the Ashadhara project has set an example for outcome-based management, ensuring equitable access to advanced treatments.

## Conclusions

The existing requirements justify the inclusion of emicizumab as an essential drug in the management of hemophilia A in the NHM’s Essential Drug List. Emicizumab, when administered as a continuous prophylactic at the appropriate dose, has been proven effective in randomized clinical trials (RCTs) and real-world settings over the past ~10 years. Notably, emicizumab is effective across all age groups and in both inhibitor and non-inhibitor patients, making it a reliable option for hemophilia A management. The WFH Guidelines, third edition, also recommend continuous prophylaxis as the treatment of hemophilia A. While FVIII plays a crucial role in treating acute bleeding episodes in PwHA, its use as a continuous prophylactic is associated with challenges. Continuous administration of FVIII can lead to the development of neutralizing antibodies, which render the treatment ineffective. Consequently, managing bleeding in patients with inhibitors requires more costly alternatives, such as rFVIIa, aPCC, or ITI. Therefore, emicizumab, with its ability to prevent joint damage and reduce bleeding episodes without the risk of inhibitor development, should be considered an essential medication for hemophilia A prophylaxis.
